# Muscle and intestinal damage in triathletes

**DOI:** 10.1371/journal.pone.0210651

**Published:** 2019-01-18

**Authors:** Łukasz Tota, Anna Piotrowska, Tomasz Pałka, Małgorzata Morawska, Wioletta Mikuľáková, Dariusz Mucha, Magdalena Żmuda-Pałka, Wanda Pilch

**Affiliations:** 1 Department of Physiology and Biochemistry, Faculty of Physical Education and Sport, University of Physical Education in Krakow, Krakow, Poland; 2 Department of Cosmetology, Faculty of Rehabilitation, University of Physical Education in Krakow, Krakow, Poland; 3 Department of Sports Medicine and Human Nutrition, Faculty of Physical Education and Sport, University of Physical Education in Krakow, Krakow, Poland; 4 Department of Physiotherapy, Faculty of Health Care, University of Presov, Presov, Slovakia; 5 Department of Biomedical Science, Faculty of Physical Education and Sport, University of Physical Education in Krakow, Krakow, Poland; 6 Department of Humanities, Faculty of Physical Education and Sport, University of Physical Education in Krakow, Krakow, Poland; Shenandoah University College of Arts and Sciences, UNITED STATES

## Abstract

The aim of the paper was to assess indicators of muscle and intestinal damage in triathletes. The study involved 15 triathletes whose objective for the season was to start in the XTERRA POLAND 2017 event (1,500-m swimming, 36-km cycling, and 10-km mountain running). Before the 14-week preparatory period, the competitors’ body composition was measured, aerobic capacity was tested (graded treadmill test) and blood samples were collected to determine markers showing the level of muscle and intestinal damage. Subsequent tests for body composition were carried out before and after the competition. Blood samples for biochemical indicators were collected the day before the competition, after the completed race, and 24 and 48 hours later. A significant decrease in body mass was observed after completing the race (–3.1±1.5%). The mean maximal oxygen uptake level among the studied athletes equalled 4.9±0.4 L·min^–1^, 58.8±4.5 mL·kg^–1^·min^–1^. The significant increase in concentrations of cortisol, c-reactive protein and myoglobin after the competition, significantly correlated with the significant increase in zonulin concentration (post 1h: *r* = 0.88, *p* = 0.007, *r* = 0,79, *p* = 0.001, *r* = 0.78, *p* = 0.001, and post 12h: *r* = 0.75, *p* = 0.01, *r* = 0.71, *p* = 0.011, *r* = 0.83, *p* = 0.02). No significant changes in the concentration of tumour necrosis factor alpha among the examined competitors were noted at following stages of the study. The results of our research showed that in order to monitor overload in the training of triathletes, useful markers reflecting the degree of muscle and intestinal damage include cortisol, testosterone, testosterone to cortisol ratio, c-reactive protein, myoglobin and zonulin. Changes in muscle cell damage markers strongly correlated with changes in zonulin concentration at particular stages of the study. Thus, one can expect that the concentrations of markers depicting the level of muscle cell damage after an intense and long-lasting effort will significantly influence the level of the intestinal barrier.

## Introduction

The triathlon is an Olympic discipline of endurance exercise that includes 1.5 km of swimming, 40 km of cycling, and 10 km of running. Top competitors need less than 2 hours to cover all these three distances [1]. Recent years have brought about an increase in participation concerning mass endurance sports events such as the triathlon. Therefore, the interest in training cycles has increased regarding this discipline. It should be noted that the periodization of the training process for competitors preparing to complete a triathlon is very complex. The variable work environment forces athletes and coaches to seek new training solutions that would strengthen the body’s adaptation to the exhausting efforts during a race. Owing to the typically endurance nature of the competition, the whole training process should be systematically modified and adjusted to the individual potential of an athlete. Therefore, optimization of training demands continuous control and monitoring of the training process with methods adjusted to the specificity of a sports discipline so that the obtained measurements provide reliable information.

Mechanical and metabolic stress resulting from the intense work of muscle cells during a long-lasting effort, constitutes a significant factor inducing damage to cell structure and function. The main reasons for this damage are increasing energetic crisis, dehydration and hyperthermia [2]. As a consequence, muscle fibre damage occurs, which leads to the release of inflammatory cytokines such as tumour necrosis factor alpha (TNF-α) [3]. Moreover, a long-lasting and exhausting physical effort can cause an increase in markers typical of cardiac muscle damage, e.g. myoglobin [4]. Observing changes in biochemical indicators under the influence of stress induced by physical exercise has been the subject of numerous studies. In the context of the undertaken sports effort, changes in the concentration of testosterone (T), cortisol (C) [5] and the c-reactive protein (CRP) [6] have been analysed.

During a long-lasting physical effort, gastrointestinal blood flow decreases to the advantage of increased activity of other organs. Research shows that athletes training mainly endurance disciplines are particularly vulnerable to the occurrence of such symptoms as contractions, abdominal pain, nausea or diarrhea during a race. These are symptoms related to intestinal permeability disorders [7]. Zonulin is evaluated in order to diagnose the degree of the intestinal damage [8].

The physiological and biochemical requirements of intense exercise induce a range of muscular and systemic reactions. Some studies have observed deterioration in intestinal permeability assessed according to changes in concentration of zonulin in response to extreme exercise-induced stress in the training of individuals after endurance and/or interval training [9, 10].

Regular and moderate physical activity leads to many beneficial functional changes related to gastrointestinal state; however, as the intensity and volume of the training units increase, the risk of intestinal microflora disorders also rises [7]. According to various statistics, approximately 70% of athletes, mainly long-distance runners, cyclists, and triathletes, complain of gastrointestinal problems during or just after competitions [11]. In addition, long-term stress, antibiotics, as well as protein- and fructose-rich diets can exacerbate intestinal microflora disorders [12]. Zonulin is most often applied to assess the degree of intestinal damage (leaks in the intestinal barrier) [3, 13]. It should be noted that the changes in its concentration in the blood may be only influenced by intense efforts, but also by incorrect diets and intestinal bacteria [8, 14].

In the subject-related literature, however, there is a lack of human studies simultaneously analysing a number of muscle cell damage markers, while considering the indicator showing the degree of intestinal damage. The degree of intestinal damage and oxidative stress, and TNF-α, have been assessed in a few papers [3], but they only emphasise the need for further research and to focus on a larger number of markers.

In articles referring to the discussed issue, changes in the concentrations of markers reflecting the degree of muscle and intestinal damage after long-lasting physical exercise are diversified, from slight increases to high concentrations [15–17].

Hence, answering the question as to whether the levels of markers reflecting the degree of muscle and intestinal damage resulting from endurance-type efforts would allow better understanding of the physiological and biochemical reactions taking place in an athlete’s body. This, in turn, may lead to optimal adjustment of training loads in the periodization of the training process. It still remains unclear if the considerable increases in the concentration of damage and inflammatory markers regarding the muscles of athletes completing extreme runs are a transient, harmless norm or, quite the opposite, if, in the long-run, they can lead to detrimental health consequences [18].

Therefore, it seems obvious that knowing the increase in the level of markers indicating the degree of muscle and intestinal damage after a long-lasting and exhausting physical effort is useful to better understand adaptation of the human body to extreme efforts.

One hypothesis assumes that changes in the concentration of the marker indicating intestinal cell damage after completing the competition are influenced by an increase in the concentration of markers indicating muscle cell damage. Within this context, the aim of the paper was to assess the increase in the concentration of indicators reflecting muscle and intestinal damage among triathletes.

## Material and methods

The study was conducted in accordance with the Declaration of Helsinki. The study protocol was approved by the bioethical committee of the regional Chamber of Physicians and Dentists. The participants were informed of the purpose and course of the study, and provided their written consent to participate in the project. They also provided valid medical examination results, which was one of the inclusion criteria for participation in cardiac stress tests. The tests were performed under the constant supervision of a sports medicine physician.

The sports event that the athletes were preparing for in the season was the XTERRA POLAND 2017 (1,500-m swimming, 36-km cycling, and 10-km mountain running). It constituted the XTERRA world championship qualifications.

The competition was held in August 2017. The mean (range), dry-bulb temperature during the event equalled 31.2±3.1°C (25–33°C), the relative humidity was 69±8% (60–71%), and the dew point totalled 21±3°C (18–25°C). The swimming part was performed in a reservoir with the water temperature of 20±1°C. At this stage of the race, all the participants were equipped with wet suits. While cycling, the competitors participating in the observation used bicycles with carbon or aluminum frames.

Prior to the start, participants were not given instructions as to at what pace they should run the race, nor were they recommended an amount of consumed liquids or products during the competition. The purpose was to avoid any influence of these factors on the final result of the competition. However, after the competition, each participant was asked to estimate the amount of liquids consumed at each nutritional point. The mean amount of liquid intake by the athletes equalled 0.7±0.3 L of water and 1.5±0.5 L of isotonic drinks.

All measurements were integrated into the preparation cycle of the XTERRA competition, lasting 16 weeks.

During the preparatory period, each participant trained for approximately 2–2.5 hours a day, 5–6 times a week. Within the 2 weeks preceding the start in the competition, the subjects did not take any medication.

### Evaluation of somatic and biochemical indicators

Body height (BH), body mass (BM), fat mass (FM) and lean body mass (LBM) were assessed in the anthropometric measurements.

Body mass and composition were determined using bioelectrical impedance analysis (BIA) via the Jawon Medical body composition analyser, model IOI 353 (Korea), whereas body height was measured with the Martin type anthropometer (USA) to the nearest mm.

Somatic and biochemical indicators were determined at the beginning of the preparatory period, i.e. 16 weeks prior to the start in the competition (baseline), approximately 2 hours before the XTERRA start (pre), immediately after completing the race (post 1h), 12 hours (post 12h) and 48 hours after the end of the race (post 48h).

Somatic and biochemical measurements on the day of the competition were performed in specially prepared zones located near the start and finish lines. After completing the race, each subject left for somatic and biochemical measurements within 1–3 minutes. The participants were instructed to avoid consuming liquids after crossing the finish line until completion of the measurements. After the tests, the competitors were given water, isotonic drinks and a regenerative meal.

The measurements before the start of the preparatory period, as well as 12 and 24 hours after the end of the competition, were carried out in the competition office.

The material for biochemical tests comprised blood taken from the ulnar vein (7 mL). This was performed by a laboratory diagnostician in accordance with binding standards. The blood was collected into Vacutainer EDTA tubes.

The following indicators were assessed for the blood: testosterone (EIA1559), cortisol (EIA-1887), c-reactive protein (EIA-1952), TNF-α (EIA-4641), myoglobin (EIA-3955), total protein (MBS2540455) and zonulin (201-12-5578).

All the indicators were measured with the enzyme-linked immunosorbent assay test (ELISA), using the DRG-type microplatelet reader (E-Liza Mat 3000, Medical Instruments GmbH, Germany).

The anabolic/catabolic balance indicator was determined on the basis of the following formula: testosterone/cortisol x100 [19].

Due to post-workout dehydration, the values of biochemical indicators determined on completion of the triathlon were corrected. The correction was performed by establishing%ΔPV using the appropriate formula:%ΔPV = -100x[(Bk-Bp)/(Bk)], where: Bp–baseline protein level determined prior to the exercise; Bk—endpoint protein level determined after exercise [20].

The Kraemer and Brown [21] formula was used to calculate the corrected values. W_sk_ = (%ΔPV.0.01.W_po_) + W_po_, where: W_sk_−corrected value, W_po_−post-exercise value.

### Evaluation of physiological indicators

The physiological indicators were assessed at the beginning of the preparatory period (baseline).

For the assessment of maximal oxygen uptake (VO_2_max) and the level of the second ventilatory threshold (VT2), a graded, treadmill test was applied (Saturn 250/100R, h/p/Cosmos, Germany).

The effort started with a 4-minute warm-up at the speed of 8 km·h^–1^, with the surface inclination angle of 1°. Then, the running velocity was increased by 1.1 km·h^–1^ every 2 minutes. The test was carried out until refusal.

In the course of the test, the following indicators were registered with an ergospirometer (Cortex MetaLyzer R3): respiratory ventilation per minute, percentage of carbon dioxide in exhaled air, oxygen uptake per minute, carbon dioxide output per minute, respiratory quotient and carbon dioxide respiratory equivalent. Heart rate (HR) during the test was measured with a sports watch (Suunto Ambit 4, Finland).

For the determination of VT2, changes were analysed in respiratory indicators occurring along with the increase in work intensity. The criteria for VT2 assessment were the following: (a) the percentage of CO_2_ in the exhaled air reached its maximal value and then decreased; (b) the CO_2_ respiratory equivalent was at its minimal value and then increased; (c) when VT2 was exceeded, a non-linear, large increase in respiratory ventilation was observed [22, 23]. The highest registered quantity was considered the VO_2_max value.

### Nutritional recommendations

Before the tests, the participating competitors underwent nutritional consultations in order to rationalise and standardise their diets. The dietary assumptions were developed in accordance with the qualitative recommendations for rational nutrition of athletes [24] and the quantitative recommendations for endurance sports competitors [25, 26]. Energy demand was set at the level of 3,500–5,000 kcal, carbohydrate supply of 6–10 g/kg body mass, protein supply of 1.2–1.7 g/kg body mass and fat supply of 1.0–1.5 g/kg body mass. The athletes were acquainted with the assumptions of the peri-workout nutrition strategy [27, 28]. They were instructed on the necessity to preserve the dietary model during the whole follow-up period (16 weeks), and to eliminate supplements that could influencebiochemical tests.

### Characteristics of the studied competitors

The study involved 15 triathlon-training athletes, aged 32.8±3.1 years, with the average training experience of 10±4.2 years. The sports level of the participants corresponded to sports classes I and II (national-level competitors).

### Methods of statistical analysis and presentation of results

The results of the study are presented as arithmetic means and standard deviations. The consistency of distribution of the evaluated indicators with normal distribution was checked using the Shapiro-Wilk test. Changes in somatic and physiological indices were evaluated with the Wilcoxon test. The relationships among the described variables were determined using Pearson’s linear correlation coefficient.Statistical analysis of the results was performed with the use of Statistica 10.0 for Windows by StatSoft.Differences among all the analysed indicators were assumed statistically significant at the level of *p*≤0.05.

## Results

Statistically significant changes between the beginning and the end of the preparatory period were observed in the level of fat tissue, expressed in% and kg (baseline—FM: 14.5±2.5kg,%F: 17.3±3.1%; *p* = 0.009, pre—FM: 11.1±3.0kg,%F: 13.7±2.6, *p* = 0.002). It is also worth noting that body mass significantly decreased immediately after the completion of the race in comparison to the measurements performed before the start of the competition. Detailed analysis of the somatic indicators is presented in Table 1.

**Table 1:** Changes of selected somatic indicators in the examined competitors.

	BM [kg]	BH [cm]	LBM [kg]	FM [kg]	%F [%]
**Baseline**	83.3±3.1	181.7±4.1	68.8±3.1	14.5±2.5	17.3±3.1
**Pre**	81.1±3.0		70.0±2.9	11.1±3.0	13.7±2.6
**Post 1h**	78.6±2.5		67.8±2.5	10.8±3.1	13.8±2.8
**p**	baseline–post 1h, *p* = 0.001; pre–post 1h, *p* = 0.01		pre–post 1h, *p* = 0.02	baseline–pre, *p* = 0.009; baseline–post 1h, *p* = 0.001	baseline–pre, *p* = 0.002; baseline–post 1h, *p* = 0.001

Baseline–assessment at the beginning of the preparatory period, Pre–measurements at the end of the preparatory period, the day before the competition, Post 1h –assessment performed directly after race completion, BM–body mass, BH–body height, LBM–lean body mass, FM–fat mass,%F–percentage of fat tissue; *p*<0.05.

Table 2 presents the values of physiological indicators characterising the maximal (MAX) and threshold (VT2) effort level in the examined competitors. The average maximal running speed achieved by the subjects during the test was 16.9+10±0.4 km·h^-1^, the average level of maximal oxygen uptake was 4.9±0.4 l·min^-1^ (58.8±4.5 mL·kg^-1^·min^-1^). At the level of the second ventilatory threshold, VO_2_ totalled 3.8±0.3 l·min^-1^, (47.6±4.0 mL·kg-^1^·min^-1^).

**Table 2:** The level of selected physiological indicators in the examined competitors.

Indicators	Effort level, baseline	
	MAX	VT2
**t [min]**	20.0±1.8	13.2±0.9
**v [km·h**^**–1**^**]**	16.9±0.4	13.1±1.1
**HR [beats·min**^**–1**^**]**	181.4±7.3	161.8±3.1
**VO**_**2**_**max [L·min**^**–1**^**]**	4.9±0.4	3.8±0.3
**VO**_**2**_**max [mL·kg**^**–1**^**·min**^**–1**^**]**	58.8±4.5	47.6±4.0
**Ve [L·min**^**–1**^**]**	167.6±15.6	97.0±10.2
**%VO**_**2**_**max**	81.0±2.5	
**%HRmax**	89.2±4.9	
**Distance [m]**	3,974.8±290.1	

Baseline–assessment at the beginning of the preparatory period, MAX–maximal indicator level, VT2 –second ventilatory threshold, t–time of work in the graded test, HR–heart rate, VO_2_ –oxygen uptake per minute: globally (L∙min^–1^) and relative to body mass (mL·min^–1^·kg^–1^), Ve–respiratory ventilation per minute [L·min^–1^].

Table 3 presents the detailed changes in the concentration of biochemical indicators for the participants during the whole follow-up period. At the beginning of the preparatory period (baseline), as well as before the competition start (pre), all the analysed indicators were within the range of the reference values. One hour after completing the competition (post 1h), the cortisol concentration increased, on average, to 467.49±112.1ng•mL (pre-post 1h, *p* = 0.001), the myoglobin concentration was 381.54±112 ng•mL (pre-post 1h, *p* = 0.001) and the concentration of zonulin totalled 89.48±25.6 ng•mL (pre-post 1h, *p* = 0.001).

**Table 3:** Changes in the concentration of biochemical indicators among the participants during the follow-up period.

Indicators	Study series					
	Baseline	Pre	Post 1h	Post 12h	Post 48h	p
**Cortisol** **[ng/mL]**	152±39.4	169.03±45.7	467.49±112.1	262.83±61.7	182.88±49.7	Pre-post 1h, *p* = 0.001 Post 1-12h, *p* = 0.001
**Testosterone** **[ng/mL]**	4.11±0.9	4.78±1.2	2.51±0.71	3.12±0.81	4.55±1.6	Pre-post 1h, *p* = 0.001
**T/C indicator**	2.70±0.6	2.98±0.9	0.55±0.1	1.45±0.4	2.44±0.5	Pre-post 1h, *p* = 0.001
**CRP** **[μg/mL]**	0.1±0.1	0.55±0.3	3.38±1.4	12.06±4.6	8.69±2.9	Pre-post 1h, *p* = 0.001 Post1-12h, p = 0.001
**TNF-α** **[pg/mL]**	4.71±0.82	4.93±1.2	7.11±3.4	5.48±1.7	5.19±1.9	NS
**Myoglobin** **[ng/mL]**	18.12±13.4	26.36±14.1	381.54±112	158.92±77.6	42.34±21.3	Pre-post 1h, *p* = 0.001 Post 1-12h, *p* = 0.001
**Total protein** **[g/L]**	69.11±5.9	70.56±8.1	80.95±10.1	69.65±9.3	70.27±9.0	Pre-post 1h, *p* = 0.001 Pre-post 12h, *p* = 0.008 Pre-post 48h, *p* = 0.001
**Zonulin** **[ng/mL]**	25.42±9.1	31.69±10.3	89.48±25.6	63.89±17.8	49.78±16.9	Pre-post 1h, *p* = 0.001 Pre-post 12h, *p* = 0.002

Baseline–assessment at the beginning of the preparatory period, Pre–assessment 24 hours before the competition, Post 1h –assessment after race completion, Post 12h –assessment 12 hours after race completion, Post 48h –assessment 48 hours after race completion, T/C indicator–the anabolic/catabolic balance indicator, CRP–c-reactive protein, TNF-α–tumour necrosis factor alpha, NS–not significant; *p*<0.05.

During the 12^th^ hour following the start (post 12h), the cortisol level decreased to 262.83±61.7 ng•mL (post 1-12h, *p* = 0.001), the concentration of CRP reached its maximum level of 12.06±4.6 μg•mL (post 1-12h, *p* = 0.001) and the zonulin concentration equalled 63.89±17.8 ng•mL (pre-post 12h *p* = 0.001). No significant TNF-α changes were observed throughout the follow-up period.

Table 4 depicts changes in the studied competitors’ plasma volume during the follow-up period. Plasma volume changes determined before and after the end of the competition (pre-post 1h) totalled -12.8±3.5%.%ΔPV determined before starting the race and during the 12^th^ hour after completing the competition was at the level of 1.3±0.4%.

**Table 4:** Change in the%ΔPV plasma volume at subsequent research stages.

	Baseline and pre	Baseline and post 12h	Baseline and post 48h	Pre and post 1h series	Pre and post 12h	Pre and post 48h
**%ΔPV**	–2.1±0.9	–0.8±0.3	–1.7±0.6	–12.8±3.5	1.3±0.4	0.4±0.2
***p***	Baseline and pre–pre and post 1h, *p* = 0.001; pre and post 1h –pre and post 12h, *p* = 0.001; pre and post 1h –pre and post 48h, *p* = 0.001					

Baseline–assessment at the beginning of the preparatory period, Pre–assessment before the competition, Post 1h –assessment directly after race completion, Post 12h –assessment 12 hours after race completion, Post 48h –assessment 48 hours after race completion; *p*<0.05.

Table 5 includes correlational results between the concentrations of markers depicting the level of muscle cell damage and zonulin concentration at subsequent research stages. There were no significant correlations between changes in testosterone and zonulin concentrations at 1h (*p* = 0.616) and 12h (*p* = 0.916), or changes in TNF-α and zonulin concentrations at 1h (*p* = 0.810) and 12h (*p* = 0.730) after the race.

**Table 5:** Correlations between the markers depicting the level of muscle cell damage and zonulin concentration at subsequent research stages.

	Post 1h		Post 12h	
	*r*	*p*	*r*	*p*
**Testosterone–zonulin**		*p* = 0.616		*p* = 0.916
**Cortisol–zonulin**	*r* = 0.88	*p* = 0.007	*r* = 0.75	*p* = 0.01
**CRP–zonulin**	*r* = 0.79	*p* = 0.001	*r* = 0.71	*p* = 0.011
**TNF-α–zonulin**		*p* = 0.810		*p* = 0.730
**Myoglobin–zonulin**	*r* = 0.78	*p* = 0.001	*r* = 0.83	*p* = 0.02

Post 1h –assessment after race completion, Post 12h –assessment 12 hours after race completion, CRP–c-reactive protein, TNF-α–tumour necrosis factor alpha; *p*<0.05

Significant correlations at 1h after the end of the triathlon were observed between the changes in cortisol and zonulin (*r* = 0.88, *p* = 0.007), CRP and zonulin (*r* = 0.79, *p* = 0.001) as well as myoglobin and zonulin (*r* = 0.78, *p* = 0.001). During the 12h following the race, significant correlations were observed between changes in cortisol and zonulin concentration (*r* = 0.75, *p* = 0.01), CRP and zonulin (*r* = 0.71, *p* = 0.011) as well as myoglobin and zonulin (*r* = 0.83, *p* = 0.02).

## Discussion

The main objective of our research was to assess the degree of muscle and intestinal damage among triathletes. The study participants were triathlon training athletes, with an average VO_2_max value of 58.8±4.5 mL•kg^–1^•min^–1^. We have noted many significant changes in the observed indicators. The average decrease in body mass after completing the competition was -3.1±1.5%. Taking dehydration accompanying exercise into consideration, we determined changes in plasma volume, which before and after finishing the competition (pre-post 1h) was at a level of -12.8±3.5%. One hour after completing the competition (post 1h), the cortisol concentration increased, on average, to 467.49±112.1 ng•mL (pre-post 1h, *p* = 0.001), the myoglobin concentration was 381.54±112 ng•mL (pre-post 1h, *p* = 0.001) and the concentration of zonulin totalled 89.48±25.6 ng•mL (pre-post 1h, *p* = 0.001).

In our research, we wanted to present a broader perspective of analyses regarding numerous biochemical indicators that can contribute to more complete optimisation of the training process in endurance disciplines. The paper is addressed to coaches, instructors, sports physicians and competitors aiming at full optimisation during planning and implementing training loads in the complex periodization of the training process in endurance sports. Understanding the “battle landscape” of a triathlon participant will allow to appropriately select training units during the preparatory period, increasing the potential of the body to adapt to exhausting work.

Body composition plays a key role in obtaining high results in endurance disciplines. In our study, the observed FM and%F levels (11.1±3.0 kg; 13.7±2.6%) were higher than values noted in the case of international class competitors (5.2±1.1; 7.8±1.6%) [29]. However, the reduction in body mass after the XTERRA race in which our subjects took part (–3.1±1.5%) turned out slightly smaller than that observed by other authors in the case of the more difficult and longer Half-Ironman competition (–3.8±1.6%) [30]. This may result from the fact that the cross version of the XTERRA race was held, and on the day of the start, the air temperature exceeded 30°C, which may have additionally affected the competitors’ dehydration.

During a long-lasting effort in an environment of elevated temperature and relative humidity (temperature 31.2±3.1°C, relative humidity 69±8%), such as during a competition, significant dehydration and a decrease in plasma volume (–12.8±3.5%) occurred despite the supply of fluids to the athletes. Studies on plasma volume changes also showed a slight decrease before the competition as compared to the beginning of the preparatory period (–2.1±0.9%). This may indicate insufficient hydration of the athletes during this period. As the human body starts to accumulate more water, adapting to each post-workout dehydration, an increase in plasma volume is then observed [31]. This adaptation was not noted in the presented study.

The triathlon is a typical endurance discipline; therefore, a high VO_2_max level and maintaining work intensity during the race with relatively high%VO_2_max values are among the numerous key physiological indicators allowing athletes to achieve better results. Moreover, the specificity of this discipline is also due to the combination of three different sports (swimming, cycling, and running) [32]. In our study, the physiological indicators of the triathletes’ potential to implement an effort of maximal intensity, such as VO_2_max [L·min^–1^; mL·kg^–1^·min^–1^], turned out lower than the mean values published by other authors with reference to elite athletes [33]. It can be observed that the best in this discipline are characterised by the ability to absorb oxygen at a level of 74–78 mL·kg^-1^·min^-1^ [34].

Many authors emphasise that endurance training puts great physiological stress on the body. One of the stress hormones is cortisol, which, responsible for many physiological functions, is associated with the rate of energy recovery after an exhausting training session or competition [35]. In the assessment of regeneration status of an athlete, apart from cortisol, testosterone level is also determined [36]. These two hormones, directly involved in the metabolism of protein are observed for the purpose of monitoring the body’s regenerative processes [19, 36–37]. Moreover, on the basis of their values, the anabolic/catabolic balance indicator is calculated. Maintaining this index value at a level of <2 proves the advantage of catabolic processes over anabolic ones, and may provide significant information regarding body overloads resulting from imbalance between workout loads and regeneration processes [19]. Monitoring these hormones during a training cycle provides feedback on the contribution of anabolic or catabolic processes in the athlete’s body. Although there are many studies showing changes in these hormones after an exhausting effort [5, 37], a broader approach to the problem of monitoring exhaustion through analysing other biomarkers is still lacking. Therefore, our study provides novel insight into monitoring the human body’s reaction to the stressful factor which is physical exercise.

The presented observation of changes in T and C concentration suggests that the time needed after an effort for the concentrations of these hormones to lower to the pre-competition level is ca. 48 hours. Nevertheless, the time needed to lower C concentration to baseline values turns out to be shorter than in the case of the T concentration, which is in line with other findings [36]. Among rugby players, T concentration dropped to baseline values after 38 hours [38], and among footballers, it stabilised after 24 hours [39]. When planning successive workout loads and monitoring C levels, one should remember that maintaining this hormone at a high level for several consecutive days is dangerous [40]. It should be noted that long-lasting aerobic training sessions in high-performance athletes inhibit the biosynthesis and secretion of T [41]. In turn, long-term lowering of T concentration, which may occur in high-performance athletes training endurance competitions, may suggest a condition similar to hypogonadism [42]. The reduced resting level of T is due to, among others, an athlete’s exposition to repeated and/or long-lasting high C concentrations [43]. Thus, observing the changes among T and C concentrations in various training cycles can provide information useful in adjusting training load intensity, as additionally emphasised by other authors [36].

High concentrations of TNF-α, an inflammatory cytokine, occur along with inflammation, injury, sepsis and some infections [44]. In the presented study, we did not observe significant changes in TNF-α concentration among the participants. Many authors emphasise that TNF-α is not a sensitive marker to be utilised in monitoring overloads in sports training [45]. It should be noted that Suzuki et al. [46] also did not observe significant changes in TNF-α concentration among competitors starting in the Ironman triathlon (3.8-km swimming, 180-km cycling, and 42.2-km running). These authors suggest utilizing other cytokines (e.g. IL-6, IL-10) in the assessment of inflammation degree caused by a long-lasting effort. In literature on the topic, various hypotheses can be found attempting to explain the lack of significant TNF-α concentration changes after an exhausting physical effort. However, it should be borne in mind that in order to induce acute inflammation, leading to a significant increase in TNF-α concentration, stronger stimuli are needed than sole muscle injury resulting from physical effort [47]. Significant changes in TNF-α concentration were observed by Ogawa et al. [48] after the implementation of a 12-week health training programme in elderly women (85.0±4.5 years). These authors suggest monitoring this indicator as one of the numerous markers of inflammatory status level, the concentration of which lowers under the influence of regular health training [48].

In monitoring exhaustion, the acute-phase protein CRP can be a significant indicator. Observing changes in CRP concentration can provide information on the level of muscle cell damage and inflammation resulting from long-lasting physical efforts. An increase in CRP concentration after physical effort is a response to the elevated cytokine level (IL-6 and TNF-α) [49]. Neubauer et al. [50] observed increased CRP concentration for 5 consecutive days after the completion of the Ironman in high-performance triathletes. Also, among high-performance race walking competitors, a 152-fold increase in CRP concentration was noted after a 246-km walk. The CRP concentration maintained high for 48 hours after the race, even if the concentration of IL-6 had already dropped to pre-workout values [51]. In the presented study, we observed the highest CRP concentration at 12 hours after the completed triathlon; the level was still elevated at 24 hours post workout.

The raised myoglobin concentrations after physical effort can result from subsequent rhabdomyolysis [52]. Many authors emphasize that the high concentration of this protein after completing the triathlon is mainly the effect of running and, more precisely, mechanical injures resulting from the impact of the foot on the ground and alternating eccentric and concentric contractions [53]. Coso et al. [54] stated that monitoring myoglobin concentrations was a useful indicator in the assessment of the level of muscle cell damage in triathletes, but only in the case of running training. In the presented study, after the completion of the whole race, we observed an increase in myoglobin concentration to the value of 381.54 ng/mL; however, we remain unable to presume which triathlon effort contributed to the phenomenon and to what degree. It is worth noting that the studied subjects performed mountain running, which could have potentially had significant impact on the high myoglobin concentration after the race.

In our research, we can exclude the impact of diet on changes in zonulin concentration during the follow-up period because all the subjects followed dietary recommendations which constituted an integral part of the project. We wish to emphasise that we observed a significant change in the integrity of the intestinal barrier and an increase of permeability, as suggested by the increase in zonulin concentration among participants after completing the race. Moreover, we detected significant correlations: the low zonulin concentration before the competition significantly correlated with lower concentrations of muscle cell damage markers (cortisol, CRP, myoglobin) after the race. In turn, in the competitors with high zonulin concentrations before the competition, we noted significantly higher concentrations of muscle cell damage biomarkers. Thus, one can expect that the concentrations of markers depicting the level of muscle cell damage after intense and long-lasting efforts will significantly influence the level of the intestinal barrier.

Nonetheless, our hypothesis demands further research among a wider group of subjects. Nonetheless, as indicated by many authors, applying multi-strain probiotics can considerably improve intestinal barrier status and favourably modulate the inflammatory response in athletes [3]. Therefore, the intake of probiotics in sports prophylaxis could become part of the basic dietary supplementation, treated as causal conduct modulating the gastrointestinal microflora. With this in mind, one can suspect that minimising the many unfavourable results of long-lasting and intense physical efforts due to gastrointestinal microflora modulation may contribute to optimising the training process in endurance disciplines.

However, in order for our assumptions to become general, standard recommendations, it is necessary to conduct a greater amount of research among a larger study group and using a broader spectrum of analysed biochemical markers.

## Conclusions

The results of the presented study indicate that to monitor overloads in triathlon training, the useful markers showing the degree of muscle and intestinal damage include cortisol, testosterone, the anabolic/catabolic balance indicator, acute-phase protein, myoglobin and zonulin. We did not observe any significant changes in the tumour necrosis factor alpha during the follow-up period.

## Supporting information

S1 Table Somatic indicators in the examined competitors. (XLSX)

S2 Table Physiological indicators. (XLSX)

S3 Table Biochemical indicators. (XLSX)
